# Complex Decisions in HIV-Related Cryptococcosis: Addressing Second Episodes of Cryptococcal Meningitis

**DOI:** 10.1007/s11904-024-00691-3

**Published:** 2024-02-24

**Authors:** Abdu Musubire, Enock Kagimu, Timothy Mugabi, David B. Meya, David R. Boulware, Nathan C. Bahr

**Affiliations:** 1 Infectious Diseases Institute, Makerere University, Kampala, Uganda; 2 Division of Infectious Diseases and International Medicine, Department of Medicine, University of Minnesota, Minneapolis, MN USA; 3 Division of Infectious Diseases, Department of Medicine, University of Kansas Medical Center, 3901 Rainbow Boulevard, Kansas City, 66160 KS USA

**Keywords:** Cryptococcal meningitis, Second episode cryptococcal meningitis, Immune reconstitution inflammatory syndrome, Cryptococcosis

## Abstract

**Purpose of Review:**

This review highlights the difficulties in diagnosing and treating persons with a prior history of cryptococcal meningitis who improve but suffer from a recurrence of symptoms. This scenario is well known to those who frequently care for patients with cryptococcal meningitis but is not well understood. We highlight major gaps in knowledge.

**Recent Findings:**

We recently summarized our experience with 28 persons with paradoxical immune reconstitution inflammatory syndrome (IRIS) and 81 persons with microbiological relapse. CD4 count and cerebrospinal fluid white blood cell count were higher in IRIS than relapse but neither was reliable enough to routinely differentiate these conditions.

**Summary:**

Second-episode cryptococcal meningitis remains a difficult clinical scenario as cryptococcal antigen, while excellent for initial diagnosis has no value in differentiating relapse of infection from other causes of recurrent symptoms. Updated research definitions are proposed and rapid, accurate diagnostic tests are urgently needed.

## Introduction/Definitions

Cryptococcal meningitis is the leading cause of meningitis in sub-Saharan Africa, leading to 19% of AIDS-related deaths [1•]. Assuming the availability of lumbar puncture (LP) and cryptococcal antigen (CrAg) testing, the initial occurrence of cryptococcal meningitis can be easily diagnosed. The CrAg lateral flow assay (LFA) by Immy, Inc. (Norman, Oklahoma, USA) is > 99% sensitive and specific in diagnosing first episodes of cryptococcal meningitis [2].

**Fig. 1:**
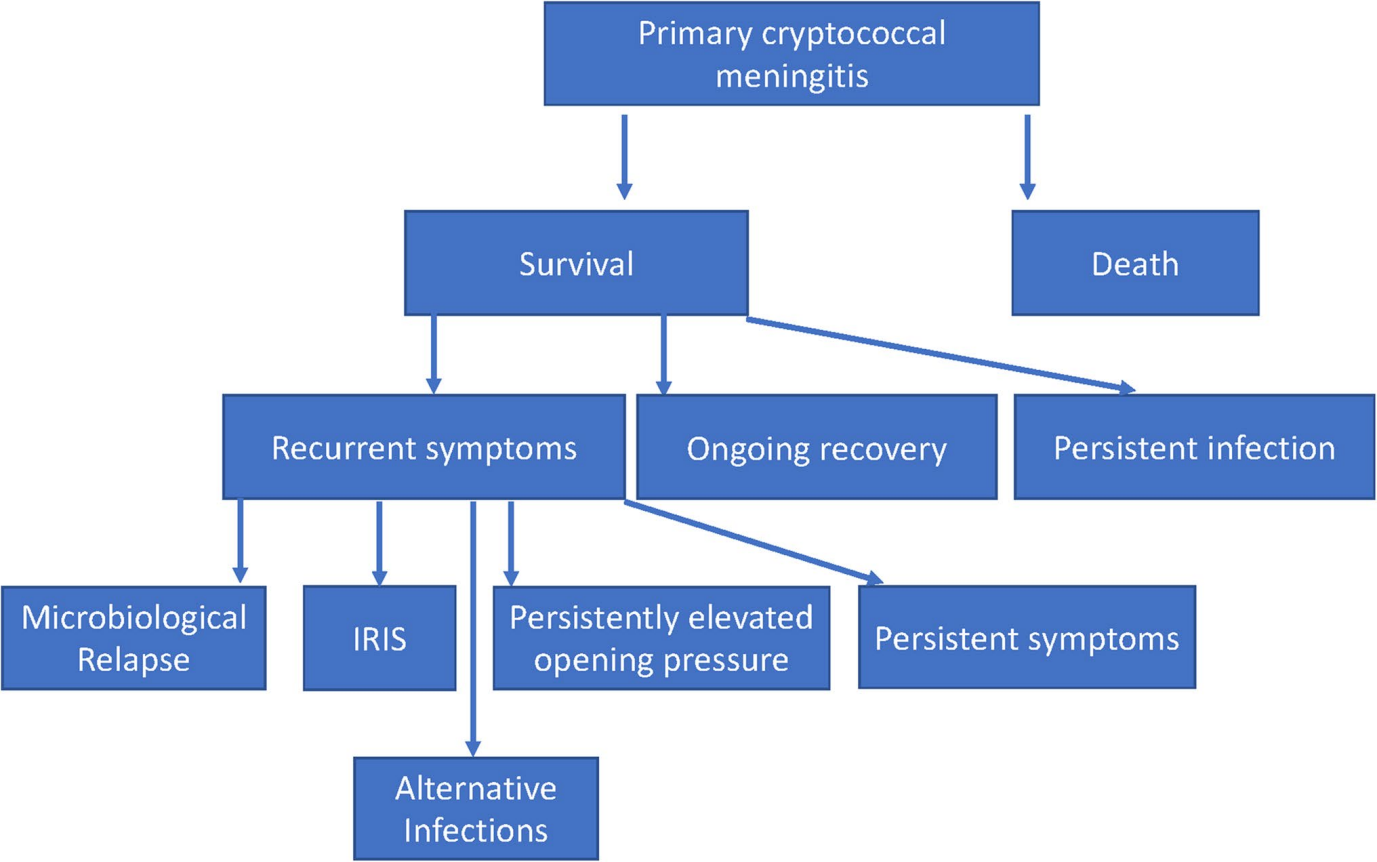
Possible etiologies of second episodes of cryptococcal meningitis. IRIS, paradoxical immune reconstitution inflammatory syndrome

While the first episode of cryptococcal meningitis can be readily diagnosed using available tools, the second episode is much more complex. When discussing a second presentation of cryptococcal meningitis, it is crucial to define the type of second presentation. A second episode indicates a scenario where a patient initially exhibited improvement after receiving treatment for cryptococcal meningitis but later experienced a recurrence of meningitis symptoms. Ideally, a negative fungal culture is available from the initial episode (from a LP conducted later in the induction course), but culture is not available in many settings. Second episodes may be due to (1) relapse of infection, (2) paradoxical immune reconstitution inflammatory syndrome (IRIS), (3) persistent elevated intracranial pressure (ICP) in the absence of IRIS or relapse, or (4) persistent symptoms of meningitis in the absence of any of the first three scenarios, Fig. 1 [3••]. Persistent infection may also present as waxing and waning symptoms, giving the impression of temporary improvement before a recurrence of symptoms. Alternative infections or co-infections are possible and should be considered; however, these are not the focus of this review [4–6].

We define these types of second presentations of cryptococcal meningitis as follows:

- Relapse: A recurrence of meningitis symptoms after initial improvement (including negative cerebrospinal fluid (CSF) fungal culture from initial episode) with positive CSF fungal culture at the time of symptom recurrence.
- Paradoxical IRIS: A recurrence of meningitis symptoms after initial improvement (including negative CSF fungal culture from initial episode) and a subsequent new start of anti-retroviral therapy (ART) OR improved ART adherence OR intervening change in ART regimen with negative CSF fungal culture at the time of symptom recurrence.
- Persistently elevated ICP: A recurrence of meningitis symptoms after initial improvement (including negative CSF fungal culture from initial episode) with negative current CSF fungal culture and persistently elevated CSF opening pressure above 250 mmH2O with improvement of symptoms on LP but no elevation in CSF cell count or protein.
- Persistent symptoms: A recurrence of meningitis symptoms after initial improvement (including negative CSF fungal culture), negative current CSF fungal culture, no elevation in CSF cell count, protein, or opening pressure.
- Persisting infection that is inadequately treated with initial induction therapy. This is usually deciphered by history of inadequate induction antifungal therapy.

A positive CSF cryptococcal culture at the time of recurrence of meningitis symptoms defines a recurrence, distinguishing this second episode from the other possibilities. Culture is the definitive test currently available, although we are aware that *Cryptococcus neoformans* can enter a viable, but non-culturable state, and so culture is likely to be imperfect in detecting all viable yeast [7]. In addition, we included CSF protein and white blood cell count in the definitions of the third and fourth categories to avoid misclassification of atypical IRIS or relapse as strictly having persistent ICP or meningitis symptoms. Although we consider these definitions to be reasonable, it is crucial to establish updated, standardized definitions that are agreed upon by key stakeholders to enable effective comparison of future research.

## The Impact of ART Availability on the Recurrence of Cryptococcal Meningitis

Given the increased availability of antiretroviral therapy, the relative proportions of populations in which cryptococcal meningitis occurs have changed. Over the last decade, the majority of patients with cryptococcal meningitis has switched from being ART-naive to having had prior exposure to ART [8–11]. Thus, in this context, an individual patient is less likely to have a new start of ART after cryptococcal meningitis than was previously the case. Rather, questions related to ART may focus on ART resistance, regimen changes, whether to continue ART that clearly has not been effective or was not being taken in an ideal fashion, and/or timing of switching regimens. All of these factors must be considered when attempting to decipher the diagnostic etiology of the second episode of cryptococcal meningitis symptoms.

## Epidemiology/Risk Factors

Cryptococcal meningitis is estimated to affect approximately 152,000 HIV-positive people annually as 2020 [1•]. Recurrent symptoms occur in approximately 5–10% of survivors [3••, 6]. As noted above, the availability of ART has increased over the past two decades which may affect the number of people presenting with a second episodes and the classification of such episodes. Before the widespread use of ART, a study conducted in Cambodia between 1999 and 2008 described 1440 people with HIV (PWH) and cryptococcal meningitis [12]. Only 2.4% (34/1440) had previously received ART at diagnosis. Of the 750 patients who were alive and in care 3 months after diagnosis, 85.9% received secondary prophylaxis for cryptococcal meningitis, and 13.7% (103/750) had a disease recurrence a median of 5.7 months after diagnosis of cryptococcal meningitis [12]. The most common type of recurrence was culture-positive relapse.

In a study conducted in Uganda after ART scale-up, we analyzed data from 724 persons with HIV-related cryptococcal meningitis of whom 16% (117/724) presented with a second episode of cryptococcal meningitis symptoms [3••]. Of the second episodes, 11% (81/724) were classified as relapse, 4% (28/724) as paradoxical IRIS, two persistently elevated intracranial pressure, and six with persistent symptoms [3••]. Among those diagnosed as relapse, 90.1% (73/81) were already on ART at presentation as were all 28 with paradoxical IRIS. Concerningly, only 53.8% (42/78) of those with available data, who were diagnosed with relapse, reported current fluconazole use. In a 2022 publication from China, paradoxical IRIS prevalence was higher, 22% (19/86) at a median of 32 days after starting ART [13].

Factors affecting the risk of relapse include (1) fluconazole monotherapy induction, which can select for resistance; (2) non-adherence to or lack of secondary prophylaxis; and (3) failure of linkage-to-care or retention-in-care of HIV ART programs [14]. Risk factors for the development of cryptococcal meningitis paradoxical IRIS include initiation of ART within 4 weeks of antifungal therapy, high initial antigen burden, and a paucity of a baseline CSF inflammatory response [15–19]. Use of fluconazole consolidation therapy at doses of 400 mg/day is also a likely significant risk factor for relapse or paradoxical IRIS, based on cross-comparison of cohorts [19–22] as is current *Cryptococcus* fluconazole susceptibility [23].

## Clinical Presentation

The clinical presentation of patients with a second episode of cryptococcal meningitis symptoms can vary, but symptoms often relate to elevated ICP or inflammation. Remarkably, persons can have CSF culture positivity without symptoms [24]; however, once increased ICP occurs, symptoms may include headache, vomiting, photophobia, or diplopia due to increased pressure on the optic nerves or glaucoma. Long-term ICP elevation may result in permanent vision loss, either due to optic nerve demyelination or secondary retinal detachment with retinal hemorrhage [25, 26]. Hearing impairment or hearing loss may be caused by inflammation of the vestibulocochlear nerve. Stiffness of the neck and lower limbs and less often backache due to irritation of the meninges or seizures can also occur, most often secondary to CSF inflammation. Other focal neurologic deficits can result from cryptococcomas. Hydrocephalus is uncommon in ART-naïve cryptococcosis, but this may occur on ART and can lead to lethargy and coma. Patients with cryptococcal relapse or IRIS may also have non-CNS manifestations of disseminated cryptococcal infection including fever, cough, shortness of breath, skin lesions, and/or lymphadenopathy [20].

The clinical presentation alone can be similar among persons with relapse, IRIS, persistent ICP elevation, persistent symptoms, or alternative infections. Patients with persistently elevated ICP often experience misdiagnosis in their second symptomatic episodes following the initiation, re-initiation, or switch of the ART regimen [14]. While relapse and IRIS are the most common etiologies, clinicians should maintain an open mind, keeping in mind the possibility of alternative infections such as tuberculous meningitis, toxoplasmosis, or CNS lymphomas, among others.

## Diagnosis

Diagnosis of second-episode of cryptococcosis is complex. The importance of taking a detailed history cannot be overstated. Information regarding the induction regimen for initial cryptococcal meningitis episode, previous/current fluconazole consolidation and secondary prophylaxis regimens (including dose and adherence), history of ART (including regimen, adherence, and timing), history of LP opening pressure measurements, and the frequency of lumbar puncture and CSF culture fungal burden during the primary episode are all potentially important details. In the absence of well-documented medical records, these details can be difficult to discern, and patients frequently will not know all of the details and complexities of their initial treatment. Further, these factors are part of the entire clinical picture and do not singularly make the diagnosis. For instance, time from initial cryptococcal meningitis episode cannot reliably differentiate IRIS from relapse. Both occur a median of 3–4 months from the initial episode with interquartile ranges in one study from 3 to 12 months [3••, 27] Testing for alternative diagnoses may be indicated but is outside of the scope of this review.

The diagnosis of primary cryptococcal meningitis can be reliably made by several tests using CSF samples obtained by LP. Although CrAg LFA is the most sensitive (> 99%), other tests such as CrAg latex agglutination (sensitivity 97%), culture (sensitivity 94%), and India ink (sensitivity related to burden) are used in different settings and in general allow for diagnosis in most cases [28].

However, antigen-based tests cannot reliably distinguish true microbiological relapse from IRIS as the cryptococcal glucuronoxylomannan polysaccharide antigen decays unpredictably, both in CSF and in blood. CrAg may persist for months to years [29–31]. Thus, after an index episode, if a patient presents with a recurrence of symptoms, CrAg will generally be positive, regardless of the cause. For instance, in our cohort of 81 patients with relapse and 28 patients with IRIS, all patients had positive CSF and blood CrAg tests [3••]. Table 1 shows performance characteristics for diagnostic tests in relapse of cryptococcal meningitis. India ink is a stain that spares the yeast cells because the *Cryptococcus* capsule cannot absorb it, while the background fill allows the viewer to visualize the *Cryptococcus* against a light background [28]. Like CrAg, India ink cannot distinguish between live and dead cells, so it cannot reliably distinguish IRIS from relapse, and the test is relatively insensitive (42% in one study < 1000 colony-forming units/mL) at low fungal burdens, meaning that cases of relapse with low burden could be missed [28, 32]. We have observed positive CSF India ink stains as long as 9 months after initial infection, in persons who were CSF culture negative with paradoxical IRIS [20]. Only culture can reliably distinguish relapse from IRIS, but results are too slow (~ 7 days) for clinical action. Thus, clinicians make empiric choices, whereby an incorrect choice means that patients are unnecessarily exposed to medication toxicities, and their actual illness is not treated.

**Table 1:** Summary of diagnostic tests for the diagnosis of cryptococcal meningitis relapse

Test	Sensitivity in relapse	Specificity in relapse	Advantages	Disadvantages	Ref
CrAg LFA	100%	~ 0%	More sensitive than culture, low cost, heat stable, rapid, and no lab infrastructure needed	Cannot distinguish between relapse and IRIS, potential false negative due to post-zone effect	[2, 3••, 28, 69]
CrAg latex agglutination	97–100%	~ 0%	Highly sensitive, relatively rapid	Less sensitive than CrAg LFA, requires cold-chain shipping, electrical supply, lab infrastructure, and labor-intensive	[17, 28]
India ink	42–86%*	0%	Rapid, cheap, and widely available	Insensitive at fungal burdens (< 1000 CFU/mL), not specific	[2, 3••, 28]
Culture	~ 94%*	100%	Current reference standard for diagnosis of relapse, quantitative culture may give clinicians information on treatment response	Slow (up to 14 days) to results, significant infrastructure and lab expertise requirements may miss non-culturable but viable yeast in relapse cases	[3••, 28]
BioFire FilmArray PCR	82%	92%	More rapidly negative on repeat testing compared to CrAg and more closely mirrors culture	Poor sensitivity at low fungal burdens (< 100 CFU/mL), high cost, and infrastructure requirements, cold-chain shipping	[3••, 33•, 34•]

*CrAg* cryptococcal antigen, *LFA* lateral flow assay, *CFU* colony-forming unit

^*^Performance data is estimated in some cases based on performance in primary cryptococcal meningitis and other factors where data specific to relapse is not available

There are novel tests which are of interest. We tested the CSF BioFire FilmArray Meningitis/Encephalitis panel (*Cryptococcus* is one of 14 pathogens detected by this multiplex polymerase chain reaction (PCR) assay). Of the 70 follow-up samples collected within a month of initial diagnosis, a negative BioFire test had 84% (26/31) negative predictive value (compared to culture), meaning when the BioFire FilmArray was negative, the CSF culture was also negative. Among two meningitis cohorts [33•, 34•], 19 persons presented with recurrent symptoms. The BioFire PCR correctly identified 11 of 12 who had a culture-positive relapse with a positive test, and 7 of 7 with paradoxical IRIS with a negative test [33•, 34•]. Similar results are presented by Van et al. [35].

Metagenomic next-generation sequencing (mNGS) is also of interest in CNS infections. In our study of second episode cryptococcal meningitis, mNGS correctly identified 10 of 11 participants with relapse, and mNGS did not detect *Cryptococcus* in 9 participants adjudicated to have paradoxical IRIS or 1 participant with isolated persistent symptoms [3••]. Although mNGS, in one study, was able to detect most cases positive by CrAg or culture, performance was poorer after exposure to antifungal therapy [36]. Yet, more data is required on both the BioFire panel and mNGS to differentiate relapse and other causes of second episode cryptococcal meningitis. Other major barriers to implementation include cost of the tests, equipment requirements, and, in the case of BioFire, poor sensitivity of only 29% (2/9) at low fungal burdens when the quantitative CSF culture < 100 CFU/mL [3••].

Thus, while BioFire PCR appears promising, its diagnostic performance in the second episode of cryptococcosis is based on fewer than two dozen cases. It remains concerning that PCR misses low-growth culture positives, which take the longest to grow. Thus, culture remains the key diagnostic test. CSF culture requires at least 100 mcL of CSF (ideally 1 mL) as an adequate input volume.

## Immunology

Fundamentally, the CSF immune response differs at time of culture-positive relapse vs. paradoxical IRIS [17, 20]. Culture-positive relapse can frequently be associated with either an anergic immune response (i.e., absence of CSF pleocytosis) or an inappropriate Type-2 T-helper cell (Th2) CD4 response with elevated interleukin-13 in CSF. Conversely, paradoxical IRIS often has an appropriate type of immune response with Type-1 T-helper cell (Th1) response with CSF elevations of interferon-gamma and chemokine CXCL10.

An example described previously of an insightful case of a research participant with culture-positive relapse at 12 weeks followed by paradoxical IRIS event at 20 weeks of ART [20]. At time of relapse, CSF grew 34,000 colony-forming units (CFU) of *Cryptococcus* per mL of CSF. The CSF white cells and protein were unchanged from initial diagnosis (5 WBCs/µL and protein 40 mg/dL). In comparison to time-matched controls without IRIS or relapse, no serum cytokine was > 1 standard deviation (SD) different from controls with only a minimally elevated serum c-reactive protein (CRP) of 10.8 mg/L. This participant’s ART was optimized, and 4 weeks later at time, they presented with paradoxical IRIS, the CRP had risen to 98.2 mg/L, and multiple pro-inflammatory serum cytokines were elevated > 3SD from the mean of time-matched controls including interleukin(IL)-1ra, IL6, GM-CSF, and to a lesser degree IL-17 and interferon-gamma (e.g., > 1SD and < 3SD elevated. In CSF, CSF white cells and interferon-gamma were markedly increased. Thus, we observed a clear immunologic difference between relapse and paradoxical IRIS, with very little inflammation evident at the time of relapse but marked inflammation present at the time of IRIS. In other cases, at time of relapse, more Th2 inflammation was observed with ~ 100-fold increased CSF levels of IL-13 in comparison to paradoxical IRIS.

## Treatment

Management of second-episode of cryptococcal meningitis depends on the final diagnosis. In addition, one must take into account the seriousness of the illness and its associated complications, as well as the patient’s ART status. A thorough history may provide important information when empiric treatment choices are necessary. Table 2 shows management strategies for the various second presentations of cryptococcal meningitis.

**Table 2:** Treatment for second-episode cryptococcal meningitis

Intracranial pressure management, antifungals, and corticosteroids	
ICP control	**A key component of cryptococcal care in all scenarios is ICP control** • Lumbar puncture performed with a manometer in the lateral decubitus position to measure CSF opening pressure • With initial elevated ICP and/or ongoing symptoms, daily lumbar puncture, until pressure is normalized • Monitor for recurrence of headache, at times twice daily lumbar punctures are necessary, initially • If no CSF opening pressure initially measured, strongly consider repeat lumbar puncture within ≤ 48 h. When a headache is present, repeat immediately
Cryptococcal meningitis relapse without complications	• ICP control with daily lumbar puncture Management according to WHO guidelines **Induction** • Single-high dose amphotericin B liposomal (10 mg/kg) AND flucytosine (100 mg/kg/day) + fluconazole 1200 mg/day for 14 days *Or* • Amphotericin B deoxycholate (1 mg/kg/day) + flucytosine (100 mg/kg/day) for 1 week, followed by 1 week of fluconazole (1200 mg/day for adults) *Or* • • Fluconazole (1200 mg daily for adults) + flucytosine (100 mg/kg/day for 14 days) **Consolidation therapy for 8 weeks** • Fluconazole 800 mg/day
Cryptococcal meningitis relapse with suspected fluconazole resistance	• ICP control with daily lumbar puncture Offer the WHO first-line induction therapy treatment as above and obtain fluconazole resistance testing if available, concurrently. If fluconazole resistance is confirmed with IC_50_ ≥ 64 µg/mL, use alternative management strategies **Induction** • Amphotericin B/liposomal + flucytosine × 7 to 14 days **Consolidation therapy** • Weekly amphotericin B deoxycholate at 1 mg/kg or liposomal amphotericin B at 3–5 mg/kg until CD4 > 200 cells/ µL *Or* Alternative azoles that may be used: • Voriconazole (400 mg every 12 h on day one then 200 mg twice daily) • Isavuconazole (200 mg every 12 h on day one then 200 mg once per day) • Posaconazole (300 mg every 12 h on day one, then daily) • Itraconazole (200 mg every 8 h times × 3 days then twice per day) Or if no options exist: • Achieve CSF sterility with amphotericin induction therapy; document sterility, then: • Monthly amphotericin B with high-dose fluconazole daily ◦ With fluconazole 800 mg/day, ~ 70% will achieve CSF concentrations > 32 µg/mL ◦ With fluconazole 1200 mg/day, ~ 85% will achieve CSF concentrations > 32 µg/mL and 50% achieve > 64 µg/mL ◦ With fluconazole 1600 mg/day, ~ 70% will achieve CSF concentrations > 64 µg/mL [23]
Cryptococcal meningitis relapse with suspected cryptococcoma	• Liposomal amphotericin 10 mg/kg every 2 weeks in combination with fluconazole 800–1200 mg daily and flucytosine 100 mg/kg/day • Monitor resolution of symptoms and reduction in the CNS lesion size with CT scans/MRI • For consolidation therapy, switch to fluconazole 800–1200 mg daily with improvement of symptoms or imaging • ICP control can be important but may need to be more cautious depending on clinical picture (e.g., focal neurological signs)
Cryptococcal meningitis relapse and suspected paradoxical IRIS	• ICP control with daily lumbar puncture • Antifungals as above for relapse • Cautious use of corticosteroids can be considered, so long as amphotericin B is being given as re-induction therapy • ICP control with daily lumbar puncture, until pressure is normalized
Paradoxical IRIS	• ICP control with daily lumbar puncture • If persistent symptoms or life threatening, corticosteroids are used. Typical dosing is 1.5 mg/kg prednisone equivalent daily with tapering after 2–4 weeks based on the patient’s response. Corticosteroid-sparing agents have been used when tapering is difficult to achieve or side effects are severe • For critically ill patients with altered mental status and inflammatory CSF with pleocytosis, higher initial corticosteroid does have been used • Serum C-reactive protein may be useful to gauge response to steroids and guide tapering
**ART management**	
ART naïve cryptococcal meningitis relapse	• ART naïve, stable patients: Initiate ART(1st line) between 4 and 6 weeks after antifungal therapy commencement • ART naïve patients with other opportunistic infections and very low CD4: May consider ART earlier after 2 weeks on a case by case basis and clinical judgement keeping in mind this may lead to worse cryptococcal meningitis outcomes and is high risk and so one must believe benefit for other OI’s is paramount
ART experienced cryptococcal meningitis relapse	• Recently started on ART < 1 month (adherent/non adherent): Hold ART-re-initiate 4 to 6 weeks later with concurrent antifungal therapy • Started on ART between 1 and 3 months (adherent/non adherent): Continue ART, manage as possible unmasking IRIS • On ART > 3 months (adherent): Continue ART, rule out treatment failure, consider ART switch after 4 to 6 weeks • On ART > 3 months, recent/ current drug interruption: Delay re-starting ART by 4 to 6 weeks, then re-initiate on current 1st line ART. Monitor viral load
**Isolated ICP elevation and complication management**	
Elevated ICP with no CNS complications	• Repeat daily therapeutic lumbar punctures until normalized on 2 consecutive days and with resolution of symptoms • Lumbar drain or VP shunt are options for persistently elevated pressure, ideal timing unclear • Patient education essential to communicate promptly if repeated headache occurs • Occasionally corticosteroids may be useful
Symptomatic communicating hydrocephalus	• VP shunt placement is preferred, in some settings frequent LP may be used as an alternative
CNS mass effect	• Variable management, may require surgery, corticosteroids • Consult with neurosurgery
**Persistent symptoms and alternative infections**	
Persistent symptoms	Symptomatic treatment, ongoing search for alternative diagnoses
Alternative infection	Treatment directed to the alternative infection, varies by pathogen

*IRIS* immune reconstitution inflammatory syndrome, *ICP* intracranial pressure, *ART* anti-retroviral therapy, *CNS* central nervous system, *VP* ventriculoperitoneal

The approach to managing relapse of cryptococcal meningitis may be altered by the level of fluconazole adherence. Many cases of cryptococcal meningitis relapse are caused by insufficient fluconazole secondary prophylaxis, ineffective or inadequate induction phase antifungal therapy, or non-adherence to antifungal medications [3••, 14]. Relapse is more likely if fluconazole has not been used as recommended. In such situations, first-line induction antifungal therapy based on the WHO’s treatment guidelines for initial episode cryptococcal meningitis is typically used [37•]. Because treatment trials specifically designed for relapse have not been done, recommendations are extrapolated from primary cryptococcal meningitis if the risk of fluconazole resistance is deemed low (low local fluconazole resistance patterns and no/minimal previous fluconazole treatment experience).

We agree with the WHO recommendations for the induction phase of cryptococcal meningitis as treatment for relapse with one of the following: (1) a single high dose of liposomal amphotericin B (10 mg/kg) AND flucytosine (100 mg/kg/day) + fluconazole 1200 mg/day for 14 days OR (2) amphotericin B deoxycholate (1 mg/kg/day) + flucytosine (100 mg/kg/day) for 1 week, followed by 1 week of fluconazole (1200 mg/day). For persons who are not ill enough to be hospitalized, one alternative outpatient regimen can be fluconazole (1200 mg daily) + flucytosine (100 mg/kg/day, divided into four doses per day for 14 days. Fourteen days of re-induction therapy are typically unnecessary and/or hospitalization for that long becomes impractical/unnecessary, thus we favor single-dose liposomal amphotericin. The induction phase is followed by consolidation and secondary prophylaxis with fluconazole. The initial approach should also include ICP management, reinforcement of adherence, fluconazole testing when indicated/possible, and delay in the start of ART for 4 weeks in those not currently on (and adhering to) ART [37•].

At time of second episode, any positive culture should have fluconazole susceptibility tested. The minimum inhibitory concentrations for fluconazole have been rising against *Cryptococcus neoformans* clinical isolates, particularly with a two-fold increase over the last decade [23, 38•, 39, 40]. Importantly, in situations where patients have strictly adhered to fluconazole treatment, fluconazole resistance may still occur, and resistance testing is even more important in such cases of treatment failure even when adherence appears to have occurred. Among persons taking fluconazole at 200 mg/day, a projected 21% will have fluconazole plasma and CSF levels below the 50% inhibitory concentration (IC_50_), and at 400 mg/day, an estimated 8.8% will have plasma concentrations below the IC_50_ [23]. While CSF and plasma have similar fluconazole concentrations, parenchymal brain tissue concentrations of fluconazole are only ~ 50% of that of plasma (unpublished). Pending susceptibility results, increasing the fluconazole dose for consolidation therapy is prudent.

In the event of fluconazole resistance, multiple studies have investigated the utilization of alternative antifungal agents [41, 42]). In vitro analysis demonstrated that itraconazole effectively eliminated fungi; however, its limited ability to penetrate CSF resulted in a higher incidence of relapse compared to fluconazole [43, 44]; although the original clinical trials were performed with tablet formulations of itraconazole which has more variable bioavailability [44]. Voriconazole has good CNS penetration, and its effect was similar to adjunctive fluconazole as part of amphotericin-based combination therapy [45, 46]. Isavuconazole, similarly has CNS penetration with some reports of use in case reports [47–49], yet, isavuconazole is not readily available in most low- and middle-income country settings. Posaconazole is also of interest [50]. These are generally only used as alternative agents for consolidation/secondary prophylaxis or as part of salvage therapy regimens. First-trimester pregnancy is also another challenging situation. We have also used weekly intravenous amphotericin, when avoidance of azoles is necessary, given the long half-life and lack of teratogenicity [51•].

### Cryptococcoma(s)

Patients who experience relapse with CNS cryptococcoma(s) usually have slower elimination of the fungus and require prolonged administration of intravenous amphotericin (sometimes up to 6 weeks) [52, 53]. In such situations, instead of prolonged daily administration for weeks, we favor liposomal amphotericin given at 10 mg/kg every 2 weeks with duration re-assessed based on clinical status in combination with fluconazole 800–1200 mg/day and ideally flucytosine 100 mg/kg/day. In this situation, the primary way to determine if it is appropriate to switch to consolidation therapy is primarily by symptoms of improvement and/or the appearance of the lesion(s) on imaging. However, it is important to note that this area is largely only supported by anecdotal evidence. The many poor outcomes largely discourage reporting of case reports or case series.

### Increased ICP

The management of increased ICP is crucial whether in relapse, IRIS, or in patients with neither but with ongoing issues with elevated intracranial pressures. WHO recommends (and we agree) that patients should undergo daily therapeutic LPs regardless of their initial opening pressure [37•]. This should continue until the pressure returns to normal for two consecutive LPs, and symptoms of increased ICP have resolved [37•]. In patients who have consistently elevated ICP, lumbar drains and ventriculoperitoneal shunts are options where available [54–57]. These interventions can decrease both the severity of the illness and the number of deaths. However, the risk of secondary infections makes these treatments less desirable unless absolutely necessary, especially in settings with limited resources [57, 58]. Mannitol and acetazolamide are ineffective in controlling ICP and may be harmful, so they are not recommended [59–61].

### Paradoxical IRIS

Treatment for paradoxical IRIS should initially focus on controlling elevated ICP through therapeutic LPs. Based on the degree of CNS inflammation present, therapy may be supportive or corticosteroids may be considered. Corticosteroids should be used in cases of life-threatening or persistent IRIS [18]. Serum CRP can be a good guide to assess response to therapy. At time of IRIS, serum CRP is often elevated (median 40 mg/L, IQR 21 to 129) which can at times reach non-physiologic extreme elevations, i.e., > 300 mg/L, in severe cases [20]. In cases of IRIS, CRP responses rapidly with corticosteroid use, and CRP can be used as a surrogate marker to guide burst then taper of steroids. For difficult, persistent cases, to spare corticosteroid use, some have used thalidomide [62, 63], lenalidomide [64], hydroxychloroquine [65], azathioprine [18], and monoclonal antibodies against tumor necrosis factor-alpha (TNF-α) such as infliximab or adalimumab [66]. The use of corticosteroids should be approached with caution as steroids have the potential to cause iatrogenic harm when ongoing infection is present [67, 68]. In general, when starting corticosteroids with a CSF culture pending < 7 days from collection, we always escalate antifungal therapy, pending culture results. Single-dose liposomal amphotericin given at 10 mg/kg can be particularly useful in this situation to provide enhanced antifungal therapy to cover for possible culture-positive relapse while initiating empiric corticosteroids for IRIS. When there is a lack of CSF pleocytosis or a lack of serum CRP elevation, one should reconsider the diagnosis of paradoxical IRIS before giving empiric corticosteroids.

### Timing of HIV Therapy Change or Re-initiation

ART initiation should be delayed by 4–6 weeks in ART-naive patients [9] or those who have stopped taking ART prior to admission, who are stable, have no other life-threatening co-morbidities, and relatively good CD4 counts above 50 cells/ µL after starting antifungal therapy. In ART-experienced patients, the management of ART is approached on an individual basis, considering factors such as adherence history, timing, and obtaining current viral load measurements before reaching a decision.

## Conclusions/Future Research

Second episodes of cryptococcal meningitis present a clinical conundrum, and a paucity of published literature exists for this condition. Herein, we have proposed standardized clinical case definitions of the common scenarios observed, which can help standardize clinical care and standardize future research.

The role of ART access is important but as access improves, cryptococcal meningitis continues to occur [10]. Thus, proper and continuous ART use, in addition to access, is crucial to cryptococcal meningitis relapse and paradoxical IRIS prevention.

Additionally, our understanding of the ideal treatments for both cryptococcal antigenemia and cryptococcal meningitis continues to evolve. It is crucial that as knowledge improves, health systems and clinicians adapt. Adherence to those standard of care treatments is important not only for patients but also for health systems and Ministries of Health (who often decide what treatments are available) as well as primary care providers. Ultimately, adherence to the best treatments is likely to prevent future relapse, IRIS, and drug resistance.

That said, even in ideal scenarios, recurrent symptoms will occur and the current situation, where we rely on culture for diagnosis, is insufficient. Our patients need a rapid and accurate test(s) to differentiate relapse from other causes of recurrent symptoms. Because treatments vary among the potential causes of symptom recurrence and can cause harm when used in the wrong setting—this is an urgent need, particularly in low-resourced, high cryptococcal disease burden settings which the scientific community must address.

## Data Availability

No datasets were generated or analyzed during the current study.

## References

[1] • Rajasingham R, Govender NP, Jordan A, et al. The global burden of HIV-associated cryptococcal infection in adults in 2020: a modelling analysis. Lancet Infect Dis. 2022;22:1748–1755. **The most up-to-date estimate of HIV-associated cryptococcal disease burden.** 10.1016/S1473-3099(22)00499-6 PMC9701154 36049486

[2] Boulware DR, Rolfes MA, Rajasingham R (2014). Multisite validation of cryptococcal antigen lateral flow assay and quantification by laser thermal contrast. Emerg Infect Dis.

[3] •• Bahr NC, Skipper CP, Huppler-Hullsiek K, et al. Recurrence of symptoms following cryptococcal meningitis: characterizing a diagnostic conundrum with multiple etiologies. Clin Infect Dis. 2023;76:1080–1087. **Large recent study charactizing those presenting with second episode cryptococcal meningitis symptoms.** 10.1093/cid/ciac853 PMC10226736 36303432

[4] Poplin V, Boulware DR, Bahr NC (2020). Methods for rapid diagnosis of meningitis etiology in adults. Biomark Med.

[5] Ellis J, Cresswell FV, Rhein J, Ssebambulidde K, Boulware DR (2018). Cryptococcal meningitis and tuberculous meningitis co-infection in HIV-infected Ugandan adults. Open Forum Infect Dis.

[6] Ellis J, Bangdiwala AS, Cresswell FV (2019). The changing epidemiology of HIV-associated adult meningitis, Uganda 2015–2017. Open Forum Infect Dis.

[7] Hommel B, Sturny-Leclère A, Volant S (2019). Cryptococcus neoformans resists to drastic conditions by switching to viable but non-culturable cell phenotype. PLoS Pathog.

[8] Jarvis JN, Lawrence DS, Meya DB (2022). Single-dose liposomal amphotericin B treatment for cryptococcal meningitis. N Engl J Med.

[9] Boulware DR, Meya DB, Muzoora C (2014). Timing of antiretroviral therapy after diagnosis of cryptococcal meningitis. N Engl J Med.

[10] Flynn AG, Meya DB, Hullsiek KH (2017). Evolving failures in the delivery of human immunodeficiency virus care: lessons from a Ugandan meningitis cohort 2006–2016. Open Forum Infect Dis.

[11] Tugume L, Semitala FC, Owachi D, Kagimu E, Kamya MR, Meya DB (2023). Clinical characteristics and morbidity among hospitalized adults with advanced HIV disease in Uganda during 'test and treat' era. PLOS Glob Public Health.

[12] Espié E, Pinoges L, Balkan S (2010). Cryptococcal meningitis in HIV-infected patients: a longitudinal study in Cambodia. Trop Med Int Health.

[13] Han X, Liu H, Wang Y (2022). A nomogram for predicting paradoxical immune reconstitution inflammatory syndrome associated with cryptococcal meningitis among HIV-infected individuals in China. AIDS Res Ther.

[14] Musubire AK, Boulware DR, Meya DB, Rhein J. Diagnosis and management of cryptococcal relapse. J AIDS Clin Res. 2013;Suppl 3(3):S3–003. 10.4172/2155-6113.s3-003. 10.4172/2155-6113.s3-003 PMC3870901 24371542

[15] Haddow LJ, Moosa MY, Mosam A, Moodley P, Parboosing R, Easterbrook PJ (2012). Incidence, clinical spectrum, risk factors and impact of HIV-associated immune reconstitution inflammatory syndrome in South Africa. PLoS ONE.

[16] Bonham S, Meya DB, Bohjanen PR, Boulware DR (2008). Biomarkers of HIV immune reconstitution inflammatory syndrome. Biomark Med.

[17] Boulware DR, Bonham SC, Meya DB (2010). Paucity of initial cerebrospinal fluid inflammation in cryptococcal meningitis is associated with subsequent immune reconstitution inflammatory syndrome. J Infect Dis.

[18] Bahr N, Boulware DR, Marais S, Scriven J, Wilkinson RJ, Meintjes G (2013). Central nervous system immune reconstitution inflammatory syndrome. Curr Infect Dis Rep.

[19] Chang CC, Dorasamy AA, Gosnell BI (2013). Clinical and mycological predictors of cryptococcosis-associated immune reconstitution inflammatory syndrome. AIDS.

[20] Boulware DR, Meya DB, Bergemann TL (2010). Clinical features and serum biomarkers in HIV immune reconstitution inflammatory syndrome after cryptococcal meningitis: a prospective cohort study. PLoS Med.

[21] Rhein J, HupplerHullsiek K, Tugume L (2019). Adjunctive sertraline for HIV-associated cryptococcal meningitis: a randomised, placebo-controlled, double-blind phase 3 trial. Lancet Infect Dis.

[22] Rolfes MA, Rhein J, Schutz C (2015). Cerebrospinal fluid culture positivity and clinical outcomes after amphotericin-based induction therapy for cryptococcal meningitis. Open Forum Infect Dis.

[23] Chesdachai S, Rajasingham R, Nicol MR, et al. Minimum inhibitory concentration distribution of fluconazole against Cryptococcus species and the fluconazole exposure prediction model. Open Forum Infect Dis. 2019;6(10). 10.1093/ofid/ofz369. 10.1093/ofid/ofz369 PMC6767974 31420668

[24] Wake RM, Britz E, Sriruttan C (2018). High cryptococcal antigen titers in blood are predictive of subclinical cryptococcal meningitis among human immunodeficiency virus-infected patients. Clin Infect Dis.

[25] De Schacht C, Smets RM, Callens S, Colebunders R (2005). Bilateral blindness after starting highly active antiretroviral treatment in a patient with HIV infection and cryptococcal meningitis. Acta Clin Belg.

[26] Oliveira EP, de Sousa BR, de Freitas JF (2023). Clinical and epidemiological characteristics of neurocryptococcosis associated with HIV in northeastern Brazil. Viruses..

[27] Bicanic T, Harrison T, Niepieklo A, Dyakopu N, Meintjes G (2006). Symptomatic relapse of HIV-associated cryptococcal meningitis after initial fluconazole monotherapy: the role of fluconazole resistance and immune reconstitution. Clin Infect Dis.

[28] Rajasingham R, Wake RM, Beyene T, Katende A, Letang E, Boulware DR. Cryptococcal meningitis diagnostics and screening in the era of point-of-care laboratory testing. J Clin Microbiol. 2019;57(1):e01238-18. 10.1128/JCM.01238-18. 10.1128/JCM.01238-18 PMC6322457 30257903

[29] Aberg JA, Watson J, Segal M, Chang LW (2000). Clinical utility of monitoring serum cryptococcal antigen (sCRAG) titers in patients with AIDS-related cryptococcal disease. HIV Clin Trials.

[30] Antinori S, Galimberti L, Magni C (2001). Cryptococcus neoformans infection in a cohort of Italian AIDS patients: natural history, early prognostic parameters, and autopsy findings. Eur J Clin Microbiol Infect Dis.

[31] Pullen MF, Kakooza F, Nalintya E (2020). Change in plasma cryptococcal antigen titer is not associated with survival among human immunodeficiency virus-infected persons receiving preemptive therapy for asymptomatic cryptococcal antigenemia. Clin Infect Dis.

[32] Song W, Shen YZ, Wang ZY (2020). Clinical features and treatment outcomes of human immunodeficiency virus-associated cryptococcal meningitis: a 2-year retrospective analysis. Chin Med J (Engl).

[33] • Bridge S, Hullsiek KH, Nerima C, et al. Evaluation of the BioFire(R) FilmArray(R) meningitis/encephalitis panel in an adult and pediatric Ugandan population. J Mycol Med. 2021;31:101170. **Recent study evaluating the BioFire Filmarray panel in cryptococcal meningitis including those with second-episode of cryptococcal meningitis symptoms.** 10.1016/j.mycmed.2021.101170 PMC9983612 34246087

[34] • Rhein J, Bahr NC, Hemmert AC, et al. Diagnostic performance of a multiplex PCR assay for meningitis in an HIV-infected population in Uganda. Diagn Microbiol Infect Dis. 2016;84:268–273. **This study provided the first evidence related to the Biofire assay's performance in second episode cryptococcal meningitis.** 10.1016/j.diagmicrobio.2015.11.017 PMC4764472 26711635

[35] Van TT, Kim TH, Butler-Wu SM (2020). Evaluation of the BioFire FilmArray meningitis/encephalitis assay for the detection of Cryptococcus neoformans/gattii. Clin Microbiol Infect.

[36] Zhang X, Lin Y, Chen H (2023). Diagnostic performance of metagenomic next-generation sequencing in central nervous system cryptococcosis using cerebrospinal fluid. Infect Drug Resist.

[37] • World Health Organization. Guidelines for diagnosing, preventing and managing cryptococcal disease among adults, adolescents and children living with HIV. Available at: https://www.who.int/publications/i/item/9789240052178. Accessed 27 June 2022. **Current WHO guidelines for treatment of cryptococcal disease.** 35797432

[38] • Bongomin F, Oladele RO, Gago S, Moore CB, Richardson MD. A systematic review of fluconazole resistance in clinical isolates of Cryptococcus species. Mycoses 2018;61:290–297. **Systematic review of fluconazole resistance in *****Cryptococcus*****.** 10.1111/myc.12747 29377368

[39] Mpoza E, Rhein J, Abassi M (2018). Emerging fluconazole resistance: implications for the management of cryptococcal meningitis. Med Mycol Case Rep.

[40] Naicker SD, Mpembe RS, Maphanga TG (2020). Decreasing fluconazole susceptibility of clinical South African Cryptococcus neoformans isolates over a decade. PLoS Negl Trop Dis.

[41] Pfaller MA, Diekema DJ, Gibbs DL (2009). Results from the ARTEMIS DISK Global Antifungal Surveillance Study, 1997 to 2007: 10.5-year analysis of susceptibilities of noncandidal yeast species to fluconazole and voriconazole determined by CLSI standardized disk diffusion testing. J Clin Microbiol.

[42] Smith KD, Achan B, Hullsiek KH (2015). Increased antifungal drug resistance in clinical isolates of Cryptococcus neoformans in Uganda. Antimicrob Agents Chemother.

[43] Chotmongkol V, Jitpimolmard S (1992). Itraconazole in the treatment of cryptococcal meningitis. J Med Assoc Thai.

[44] van der Horst CM, Saag MS, Cloud GA (1997). Treatment of cryptococcal meningitis associated with the acquired immunodeficiency syndrome. National Institute of Allergy and Infectious Diseases Mycoses Study Group and AIDS Clinical Trials Group. N Engl J Med.

[45] Loyse A, Wilson D, Meintjes G (2012). Comparison of the early fungicidal activity of high-dose fluconazole, voriconazole, and flucytosine as second-line drugs given in combination with amphotericin B for the treatment of HIV-associated cryptococcal meningitis. Clin Infect Dis.

[46] Zhao T, Xu X, Wu Y (2022). Comparison of amphotericin B deoxycholate in combination with either flucytosine or fluconazole, and voriconazole plus flucytosine for the treatment of HIV-associated cryptococcal meningitis: a prospective multicenter study in China. BMC Infect Dis.

[47] Thompson GR, Rendon A, Ribeiro Dos Santos R (2016). Isavuconazole treatment of cryptococcosis and dimorphic mycoses. Clin Infect Dis.

[48] O'Kelly B, Mohamed A, Bergin C, et al. Successful treatment of cryptococcal meningitis and cryptococcoma with isavuconazole in a patient living with HIV. J Fungi (Basel). 2021;7(6):425. 10.3390/jof7060425. 10.3390/jof7060425 PMC8228186 34071211

[49] Ramanzini LG, de Medeiros SDP, Lima L (2023). Cerebral cryptococcoma successfully treated by isavuconazole in an immunocompetent patient: a case report. Radiol Case Rep.

[50] Kumar A, Udayakumaran S, Sachu A (2022). Ventriculoperitoneal shunt infection by Cryptococcus neoformans sensu stricto: case report and literature review. Rev Iberoam Micol.

[51] • Pastick KA, Nalintya E, Tugume L, et al. Cryptococcosis in pregnancy and the postpartum period: case series and systematic review with recommendations for management. Med Mycol. 2020;58:282–292. **Systematic review of cryptococcosis in pregnancy.** 10.1093/mmy/myz084 PMC7179752 31689712

[52] Chastain DB, Rao A, Yaseyyedi A, Henao-Martínez AF, Borges T, Franco-Paredes C. Cerebral cryptococcomas: a systematic scoping review of available evidence to facilitate diagnosis and treatment. Pathogens. 2022;11(2):205. 10.3390/pathogens11020205. 10.3390/pathogens11020205 PMC8879191 35215148

[53] Perfect JR, Dismukes WE, Dromer F (2010). Clinical practice guidelines for the management of cryptococcal disease: 2010 update by the infectious diseases society of america. Clin Infect Dis.

[54] Wen J, Yin R, Chang J (2022). Short-term and long-term outcomes in patients with cryptococcal meningitis after ventriculoperitoneal shunt placement. Front Neurol.

[55] Chai Z, Shou Y, Mungur R, Gong J, Zheng P, Zheng J (2022). Analysis of the efficacy and related factors of ventriculoperitoneal shunt for AIDS with cryptococcal meningitis. Front Surg.

[56] Li M, Liu J, Deng X (2020). Triple therapy combined with ventriculoperitoneal shunts can improve neurological function and shorten hospitalization time in non-HIV cryptococcal meningitis patients with increased intracranial pressure. BMC Infect Dis.

[57] Liu Y, Peng X, Weng W, Zhu J, Cao H, Xie S (2019). Efficacy of ventriculoperitoneal shunting in patients with cryptococcal meningitis with intracranial hypertension. Int J Infect Dis.

[58] Woodworth GF, McGirt MJ, Williams MA, Rigamonti D. The use of ventriculoperitoneal shunts for uncontrollable intracranial hypertension without ventriculomegally secondary to HIV-associated cryptococcal meningitis. Surg Neurol. 2005;63:529–31; discussion 31–2. 10.1016/j.surneu.2004.08.069 15936373

[59] Newton PN, le Thai H, Tip NQ (2002). A randomized, double-blind, placebo-controlled trial of acetazolamide for the treatment of elevated intracranial pressure in cryptococcal meningitis. Clin Infect Dis.

[60] Rigi M, Khan K, Smith SV, Suleiman AO, Lee AG (2017). Evaluation and management of the swollen optic disk in cryptococcal meningitis. Surv Ophthalmol.

[61] Srichatrapimuk S, Sungkanuparph S (2016). Integrated therapy for HIV and cryptococcosis. AIDS Res Ther.

[62] Qi T, Chen F, Ma S (2023). Thalidomide for recurrence of symptoms following HIV-associated cryptococcal meningitis. Infect Dis Ther.

[63] Brunel AS, Reynes J, Tuaillon E (2012). Thalidomide for steroid-dependent immune reconstitution inflammatory syndromes during AIDS. AIDS.

[64] Wan Z, Tao R, Hui J (2023). Efficacy and safety of lenalidomide in HIV-associated cryptococcal meningitis patients with persistent intracranial inflammation: an open-label, single-arm, prospective interventional study. J Neuroinflammation.

[65] Narayanan S, Banerjee C, Holt PA (2011). Cryptococcal immune reconstitution syndrome during steroid withdrawal treated with hydroxychloroquine. Int J Infect Dis.

[66] Sitapati AM, Kao CL, Cachay ER, Masoumi H, Wallis RS, Mathews WC (2010). Treatment of HIV-related inflammatory cerebral cryptococcoma with adalimumab. Clin Infect Dis.

[67] Beardsley J, Wolbers M, Kibengo FM (2016). Adjunctive dexamethasone in HIV-associated cryptococcal meningitis. N Engl J Med.

[68] Beardsley J, Hoang NLT, Kibengo FM (2019). Do intracerebral cytokine responses explain the harmful effects of dexamethasone in human immunodeficiency virus-associated cryptococcal meningitis?. Clin Infect Dis.

[69] Rutakingirwa MK, Kiiza TK, Rhein J (2020). "False negative" CSF cryptococcal antigen with clinical meningitis: case reports and review of literature. Med Mycol Case Rep.

